# Single-incision laparoscopic gastropexy for mesentero-axial gastric volvulus

**DOI:** 10.1186/s40792-019-0574-0

**Published:** 2019-02-04

**Authors:** Toshiaki Takahashi, Masaya Yamoto, Akiyoshi Nomura, Kei Ooyama, Akinori Sekioka, Yutaka Yamada, Koji Fukumoto, Naoto Urushihara

**Affiliations:** Department of Pediatric Surgery, Shizuoka Children’s Hospital, 860 Urushiyama, Aoi, Shizuoka City, Shizuoka 420-8660 Japan

**Keywords:** Laparoscopic gastropexy, Mesentero-axial gastric volvulus, Single incision

## Abstract

**Background:**

Mesentero-axial gastric volvulus (MAGV) is a rare but critical condition as delay in treatment can lead to lethal situations. Although the report of the surgical treatment with laparoscopic approach for MAGV has recently come to be seen, no standard procedures have been established. We aim to describe our operative technique of single-incision laparoscopic gastropexy (SILG) for MAGV and review the relevant literature to ascertain the most appropriate treatment option in these patients.

**Case presentation:**

Three patients were referred to our hospital because abdominal pain and vomiting suddenly occurred. Acute MAGV was diagnosed by upper gastrointestinal study. After overnight gastric decompression with a nasal tube, the GV was resolved spontaneously. Elective SILG was planned. Single incision at the umbilicus was made and three 5-mm trocars were inserted. The anterior wall of the body of the stomach was sutured to the peritoneum using 6 × 4-0 non-absorbable sutures for prevention of recurrence of GV and occurrence of internal hernia through the space created between the sutures. Two boys and a girl with mean age 4 ± 2 years underwent SILG. The mean time of the operation was 48 ± 23 min. All of the procedures were completed safely, and there were no postoperative complications. The mean time of postoperative hospitalization was 4 ± 1 days. All patients had good cosmetic and clinical results.

**Conclusion:**

We found SILG is a safe, technically feasible, and minimally invasive approach with low incidence of postoperative complication and the best cosmetic result for the patients with MAGV.

## Background

Gastric volvulus (GV) is a rare condition, which is defined as an abnormal rotation of the stomach of more than 180° and can be categorized into three forms, organo-axial, mesenterico-axial, and combined [1]. The absence or loosening of gastrocolic and gastrosplenic ligaments was reported to cause GV, creating a closed-loop obstruction, resulting finally in incarceration and strangulation [1].

Delays in diagnosis of acute mesentero-axial gastric volvulus (MAGV) in children can result in gastric ischemia and perforation [2, 3]. Prompt decompression and gastropexy are required to prevent recurrence [3]. Recently, the endoscopy and the laparoscopy for treatment of this condition have been reported [4–6]. Although the report of the surgical treatment with laparoscopic approach for MAGV has recently come to be seen, no standard procedures have been established.

Single-incision laparoscopic surgery (SILS) has become a popular optional procedure linking the standard laparoscopic surgery. SILS has been now applied for various operations [7–10].

We aim to describe our operative technique of single-incision laparoscopic gastropexy (SILG) for MAGV and review the relevant literature to ascertain the most appropriate treatment option in these patients.

## Case presentation

### Patients

Three patients were referred to our hospital as they suddenly have abdominal pain and vomiting. All of them had multiple episodes of recurrent same symptoms. Acute MAGV was diagnosed by upper gastrointestinal study. After overnight gastric decompression with a nasal tube, the GV was resolved spontaneously. Elective SILG was planned.

### Operative procedures

**Fig. 1:**
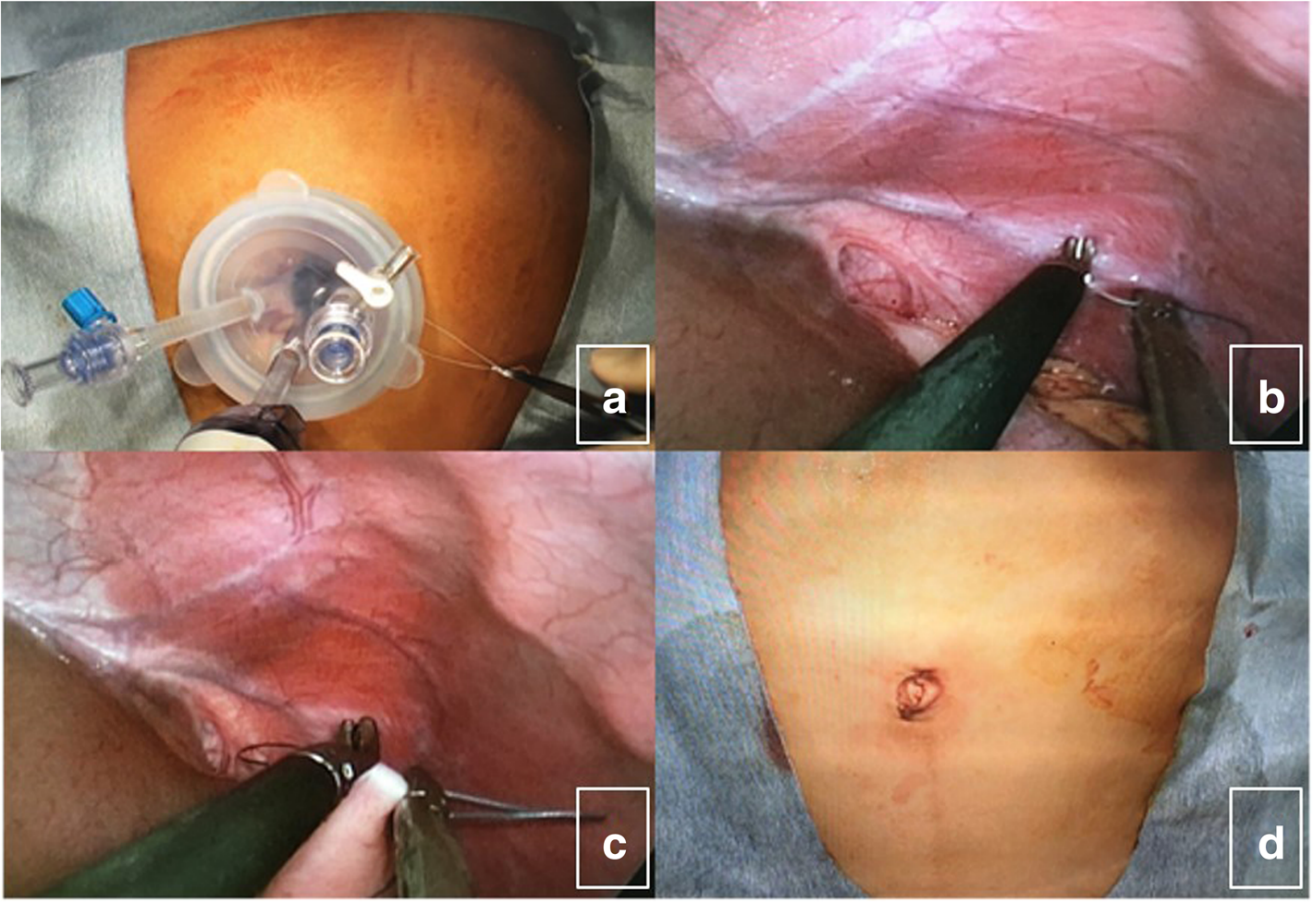
The wound retractor (Lap Protector®, Hakko Medical, Japan) was inserted, and the silicon cap (EZ access®, Hakko Medical, Japan) was mounted to the retractor. Three 5-mm trocars (EZ trocar®, Hakko Medical, Japan) were placed in the cap (**a**). After creating a pneumoperitoneum of 6–10 mmHg, a 5-mm, 30° laparoscope was inserted into one of those trocars. Then, laparoscopic exploration confirmed the presence of the gastrosplenic ligaments in all three cases. The anterior wall of the body of the stomach was sutured to the peritoneum using 3-0 non-absorbable sutures (**b**, **c**). The first suture was made on the fundus to the diaphragm. The other five sutures were made along the major curve of the stomach. We tried not to create the big space between the sutures for prevention of occurrence of an internal hernia. The scar was very small, and the patients had good cosmetic results (**d**)

During the operation, the patients were placed in a lithotomy position under general anesthesia. First, a 20-mm single vertical umbilical incision was made. The wound retractor (Lap Protector®, Hakko Medical, Japan) was inserted, and the silicon cap (EZ access®, Hakko Medical, Japan) was mounted to the retractor. Three 5-mm trocars (EZ trocar®, Hakko Medical, Japan) were placed in the cap (Fig. 1a). After creating a pneumoperitoneum of 6–10 mmHg, a 5-mm, 30° laparoscope was inserted into one of those trocars. Then, laparoscopic exploration confirmed the presence of the gastrosplenic ligaments in all three cases. The anterior wall of the body of the stomach was sutured to the peritoneum using ETHIBOND, TF 13 mm, 4-0 gauge, 75 cm, which is available for 5-mm trocars. We made all of the sutures on the left side of the round ligament of the liver. In addition, we made the knot tying of the sutures extracorporeally (Fig. 1b, c). First suture was made on the fundus to the diaphragm. Other five sutures were made along the major curve of the stomach. We tried not to create the big space between the sutures for prevention of occurrence of internal hernia. The interval of the sutures was less than about 3 cm, although it depends on the patient’s age and height. The scar was very small and the patients had good cosmetic results (Fig. 1d).

### Results

Table 1 shows the patient demographics and the operative information. Two boys and a girl with mean age 4 ± 2 years underwent SILG. The mean time of the operation was 48 ± 23 min. All of the procedures were completed safely, and there were no postoperative complications. The mean time of postoperative hospitalization was 4 ± 1 days. All patients had good cosmetic and clinical results.

**Table 1:** The patient demographics and the operative information

Case	Age (years)	Gender	OT (min)	IOC	OI (days)	POC	HS (days)
1	6	M	75	–	1	–	5
2	4	M	36	–	1	–	7
3	2	F	32	–	1	–	6

*OT* operation time, *IOC* intra-operative complication, *OI* oral intake, *POC* postoperative complication, *HS* hospital stay

## Discussion

GV is a rare condition and Berti first described in 1866 [11]. GV is defined as a rotation of all or part of the stomach through more than 180° [1]. This rotation can happen on its longitudinal (organo-axial) or transverse (mesentero-axial) axis. This condition can cause a closed-loop obstruction or strangulation. The absence or loosening of gastrocolic and gastrosplenic ligaments was reported to lead to GV [1]. Congenital diaphragmatic hernia, para-oesophageal hernia, or wondering spleen are the main secondary causes of this condition [12, 13].

In 1968, various methods of surgical repair for GV were described by Tanner [14]. These included gastrostomy, simple gastropexy, or other procedures. Most of these have been replaced with less invasive techniques. Endoscopic derotation of the stomach has given satisfactory results [5, 15]. PEG tube placement has also been reported to have success [16]. Laparoscopy has the advantage because we can see the suture placement with three dimensions [4, 6, 13, 17]. Although the report of the surgical treatment with a laparoscopic approach for MAGV has recently come to be seen, no standard procedures have been established.

**Fig. 2:**
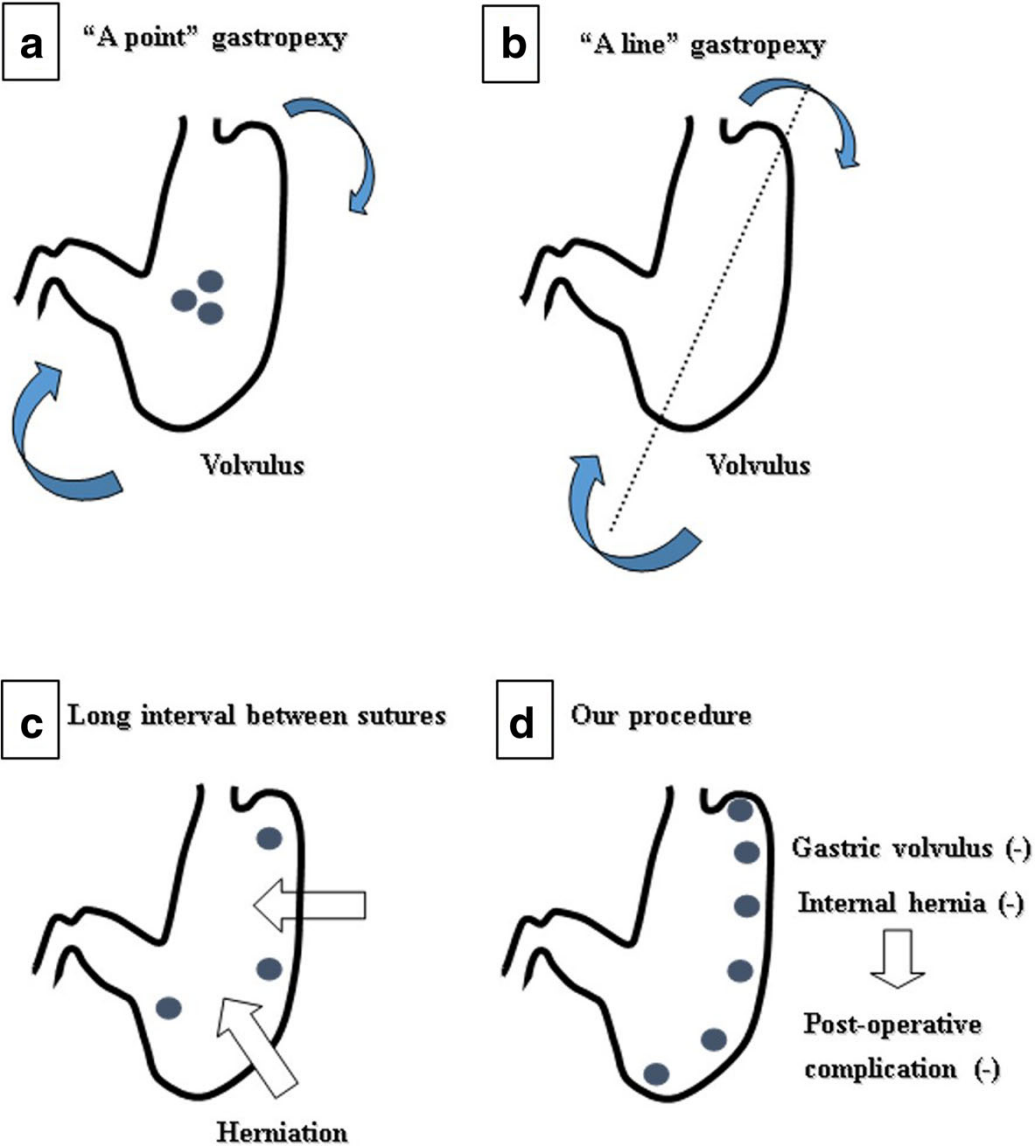
This figure shows the complication and the concept we need to take care. “A point” gastropexy (**a**) or “A line” gastropexy (**b**) may cause the gastric volvulus along the new axis which was made by a point or short range suture or a line suture. Furthermore, when we made the rough interval sutures which have big space between the sutures, an internal hernia can occur between the sutures (**c**). In our technique, the anterior wall of the body of the stomach was sutured to the peritoneum using six sutures in total for prevention of recurrence of gastric volvulus and occurrence of an internal hernia through the space created between the sutures (**d**)

Although the gastrostomy is still widely performed for the fixation of the MAGV in some institutions, there are some reports that the stomach may rotate with the gastrostomy as a new axis [18]. Another reports demonstrated that 2-3 sutures in a narrow range of the stomach may also rotate with the sutures as a new axis [19]. However, it has also been demonstrated that the big space between the sutures may allow the intestine to herniate into those space [20]. Therefore, “A point” gastropexy (Fig. 2a) or “A line” gastropexy (Fig. 2b) may cause the GV along the new axis which was made by a point or short-range suture or a line suture. Furthermore, when we made the rough interval sutures which have big space between the sutures, internal hernia (IH) can occur (Fig. 2c). In our technique, the anterior wall of the body of the stomach was sutured to the peritoneum using in total six sutures to prevent recurrence of GV and occurrence of IH through the space created between the sutures (Fig. 2d).

In addition, the cosmetic results have become more important in recent years. SILS has become a popular optional procedure and has been now employed for various operations. The conventional gastrostomy does not give the good cosmesis. Therefore, we described our procedure of the laparoscopic gastropexy by a single incision and all patients had good cosmetic as well as clinical results. However, a disadvantage point of this single-incision procedure is that the procedure might be complicated for trainees. There are some recent reports about “two-site procedure” which add one puncture to conventional SILS procedure. The two-site procedure for appendectomy has been demonstrated to be easier than SILS and not inferior to SILS from the cosmetic point of view [21, 22]. Although our cases have been performed without complications and we believe that SILG is a safe and technically feasible procedure, further studies comparing such a new procedure, the two-site procedure to our procedure, SILG, are warranted.

## Conclusion

In conclusion, we found SILG is a safe, technically feasible, and minimally invasive approach with a low incidence of postoperative complication and the best cosmetic result for the patients with MAGV.

## Abbreviations

GV: Gastric volvulus
MAGV: Mesentero-axial gastric volvulus
SILG: Single-incision laparoscopic gastropexy
